# The Clinical, Radiological, and Histopathological Characteristics of Intramuscular Myxoma: A Case Report

**DOI:** 10.7759/cureus.63144

**Published:** 2024-06-25

**Authors:** Aboubacar Lawan Abdou, Mohammed Barrached, Adnane Lachkar, Najib Abdeljaouad, Hicham Yacoubi

**Affiliations:** 1 Traumatology and Orthopedics, Faculty of Medicine and Pharmacy of Oujda, Mohammed First University, Oujda, MAR; 2 Traumatology and Orthopedics, Mohammed VI University Hospital, Oujda, MAR

**Keywords:** surgery, biopsy, mri, thigh mass, intramuscular myxoma, benign tumor

## Abstract

In this study, we discuss the clinical, radiological, and histopathological characteristics of intramuscular myxomas (IMM), a rare form of benign soft tissue tumors. We report the case of a 47-year-old female patient presenting with a painless, non-inflammatory mass in the right thigh, which was mobile relative to both superficial and deep planes. Imaging, biopsy, and subsequent histopathological study established the diagnosis of intramuscular myxoma. The patient underwent surgical excision of the mass, with a straightforward postoperative course. It is important to distinguish IMM from malignant tumors, such as soft tissue sarcomas, through comprehensive examinations including imaging and biopsy. The recommended treatment is surgery for complete excision of the mass, with an exceptionally low recurrence rate.

## Introduction

Intramuscular myxomas (IMM) are rare benign tumors affecting the soft tissue of mesenchymal origin [1]. They were first described in 1965 by Enzinger [2]. Their incidence varies between 0.1 and 0.3 per 100,000 individuals and is often seen in females aged between 40 and 70 years [1]. The most common location is in the muscles of the thigh, with less frequent occurrences in the shoulder, buttocks, leg, and arm [3,4]. The diagnosis is suggested on the history and clinical examination and is confirmed on histology. Surgical excision is the treatment of choice, with a very low recurrence rate [3].

Herein, we present a case of a patient with a myxoma located in the right thigh. Through this case, we will discuss the symptomatology, diagnostic methods, and therapeutic options of this pathological entity.

## Case presentation

The case involves a 47-year-old female residing in a rural area who presented to our clinic with swelling in her right thigh. According to her history, the swelling appeared spontaneously on the anteromedial aspect of the right thigh. Its onset was gradual, painless, and devoid of inflammatory signs or systemic symptoms. Physical examination revealed a sizable mass measuring 10 centimeters along its longest axis on the anteromedial aspect of the right thigh. Palpation revealed a non-tender, non-pulsatile, resilient mass that was mobile in relation to both superficial and deep planes. No abnormalities were noted in the inguinal lymph nodes or elsewhere during somatic examination.

**Figure 1:**
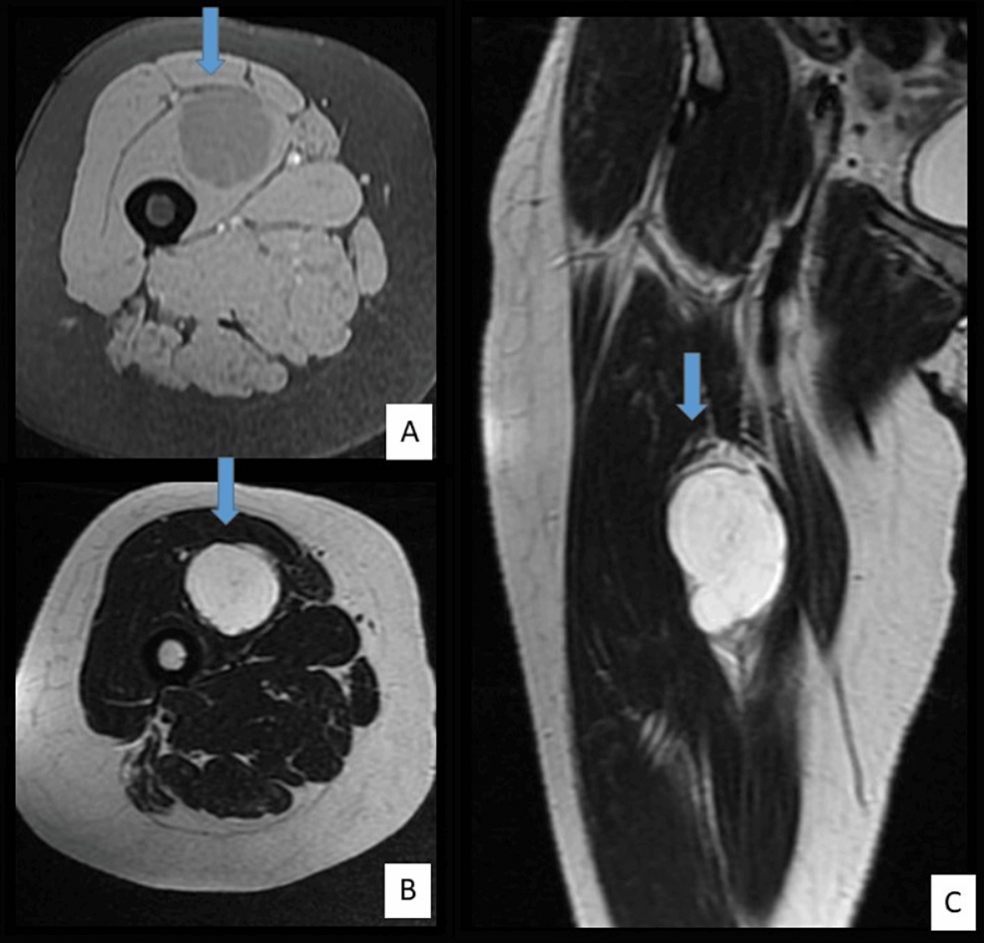
MRI images of the right thigh; A: Axial T1-weighted image showing the well-defined tumor in hypointensity. B: Axial T2-weighted image showing the well-defined tumor in hyperintensity. C: Frontal T2-weighted image showing the tumor in hyperintensity and well-defined.

The patient underwent radiological assessment, including standard X-ray imaging and MRI of the thigh, as well as laboratory tests (complete blood count with platelets, hemostasis assessment, full blood electrolyte panel including urea and creatinine, C-reactive protein). All biological test results were normal. The X-ray showed no remarkable findings. MRI findings delineated a rounded intramuscular formation arising from the right vastus intermedius muscle, with a lobulated appearance and a well-defined, thin capsule exhibiting low signal intensity on T2-weighted images, high signal intensity on T2-weighted images, isointensity on T1-weighted images, and no diffusion restriction. The mass contained thick septations, which enhanced upon gadolinium injection. Its dimensions were measured at 37x35x50 millimeters. The MRI report concluded the presence of an intramuscular mass within the right vastus intermedius muscle, displaying myxoid signal characteristics (Figure 1).

**Figure 2:**
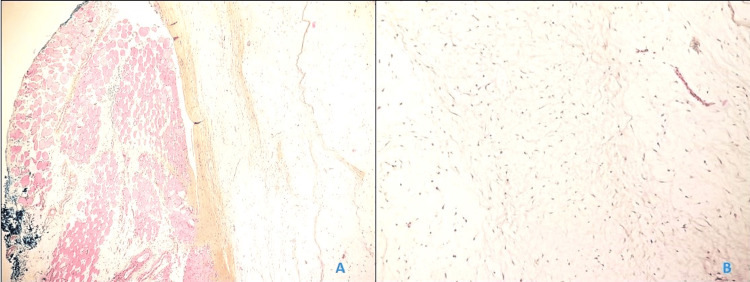
Microscopic images showing: (A) Low-power view of an intramuscular myxoma. (B) The tumor is composed of spindle-shaped and stellate cells resting on a myxoid stroma.

A biopsy was performed by the treating surgeon, yielding gelatinous tissue with a heterogeneous appearance. Microscopic examination revealed a benign tumor proliferation with low cellular density, composed of sparse cells immersed in a myxoid stroma with loose collagenous areas, devoid of tumor necrosis or malignant features. Special staining with alcian blue confirmed the myxoid nature of the proliferation. Histopathological analysis confirmed the diagnosis of intramuscular myxoma (Figure 2).

**Figure 3:**
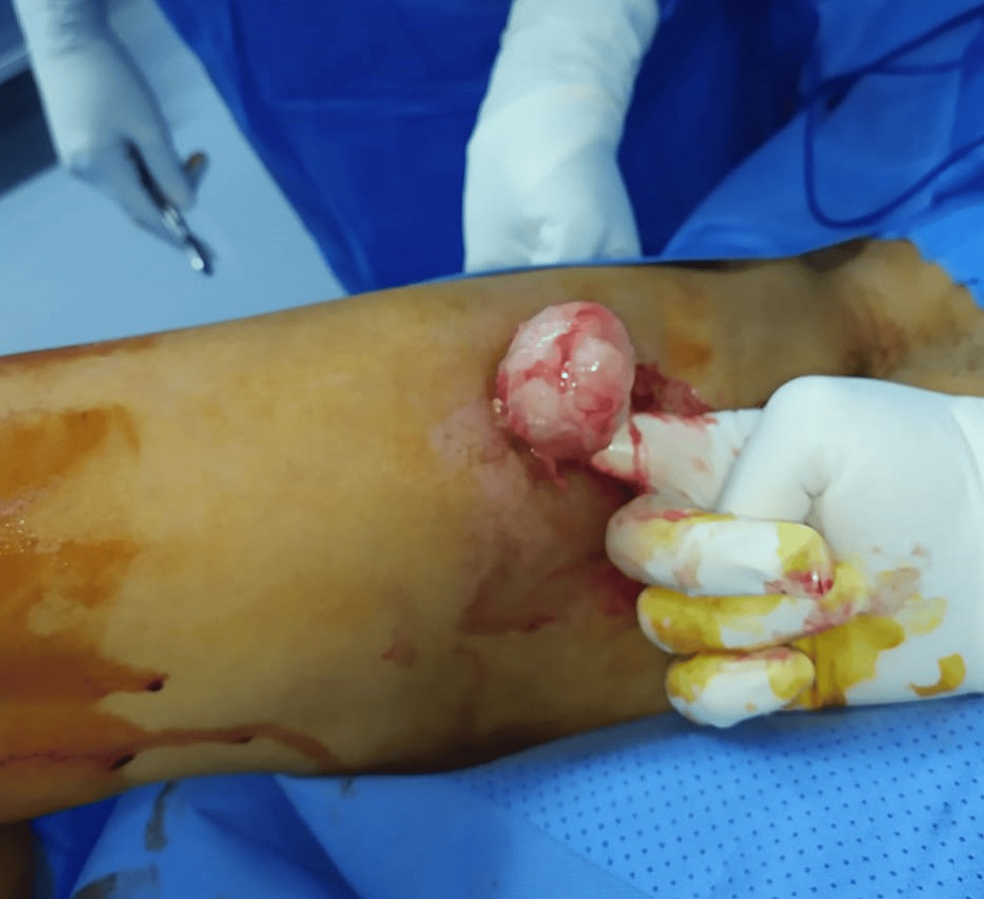
Intraoperative image of the surgical resection of the intramuscular myxoma showing a homogeneous gelatinous appearance.

**Figure 4:**
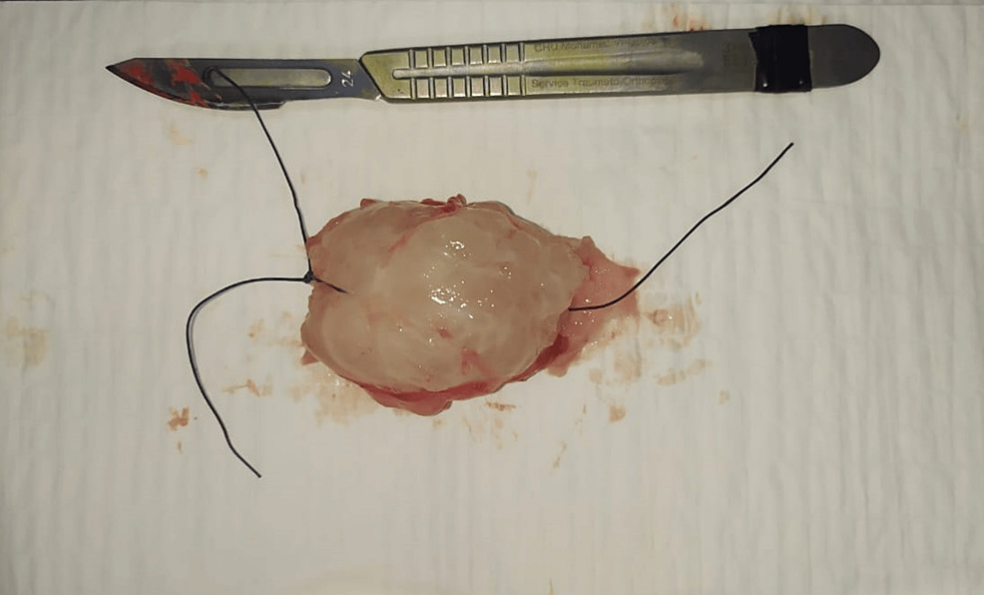
Appearance of the resected and oriented myxoma: the two threads specify the upper pole while the single thread orients the deep aspect.

The patient underwent surgical excision of the mass. Following an incision centered on the mass, encompassing the biopsy scar, a dissection plane was identified, and the mass was removed as a single block and sent for histopathological examination (Figures 3, 4).

## Discussion

IMM represent a rare form of benign soft tissue tumors, of mesenchymal origin, manifesting as deep masses. Typically described as lesions with low cellular density and reduced vascularity, with significant accumulation of extracellular myxoid matrix, these tumors can exhibit increased cellularity and vascularity with intramuscular localization, which can sometimes lead to a misdiagnosis of sarcoma. Patients usually report swelling, which occurs in regions rich in large muscles such as thighs, shoulders, arms, and buttocks [1].

IMM can be part of a clinical picture known as "Mazabraud syndrome." It describes one or more myxomas associated with fibrous dysplasia of the bone. The etiology is believed to be genetic [5,6].

Diagnosis is based on history, a thorough physical examination, imaging data, and most importantly, histology which provides certainty. History reveals the onset of swelling in areas with large muscles: thighs, shoulders, arms, and buttocks [1]. Some authors report overweight in these patients without considering it a determining risk factor [2,7]. Tumor growth is slow, with mild or no symptoms. Pain and disturbances occur only when the tumor size increases and neighboring structures are irritated or compressed [8]. This pain can be the main symptom in some patients and presents as groin pain for localization in the thigh adductors independent of the mass syndrome [9].

Our patient presented with a slowly evolving mass for 12 months, painless and without vascular or neurological signs.

MRI is performed in all patients unless contraindicated. Typically, on T1-weighted sequences, IMM appear hypointense, and on T2-weighted sequences hyperintense with enhancement after contrast agent injection. The mass is well-demarcated, surrounded by edema and cystic and/or fluid-filled areas [10].

If requested, a CT scan describes a well-demarcated mass, hyperdense at the periphery, and a hypodense center with non-homogeneous internal structures [2].

On ultrasound, a solid mass with cystic and gelatinous parts surrounded by a solid capsule is observed, with heterogeneous echogenicity [8,11].

Despite these radiological characteristics, it is reported in the literature that radiologists do not consistently consider the diagnosis of intramuscular myxoma. They often lean toward diagnosing a malignant tumor such as soft tissue sarcoma [12]. Schwannomas, liposarcoma, fibroma, myxofibrosarcoma, and hematoma are also differential diagnoses based on clinical and radiological appearance [2].

Open biopsy confirms or establishes the diagnosis of intramuscular myxoma whenever performed. Needle aspiration, on the other hand, is prone to false negatives and should therefore be avoided [13,14]. The surgeon must communicate clinical and paraclinical information to the pathologist, who is obligated not to yield in cases of insufficient or poor-quality material [15].

The treatment of choice is surgery consisting of complete excision of the mass. Recurrences are rare [3,4,16]. Resection margins are recommended only in the absence of a biopsy, avoiding further surgery in the unlikely event of a malignant tumor [2]. Recurrences are very rare and occur in patients with incomplete excision and high cellularity lesions [16].

Macroscopically, the color varies from translucent beige to gray. The mass appears well-demarcated, rounded, and multilobed with a gelatinous texture [15].

Histological examination of the resected specimen is mandatory even if the pathological analysis was definitive regarding the diagnosis of IMM [17].

## Conclusions

IMM is a benign lesion despite its potential for rapid growth. The main challenge is to ensure the exclusion of differential diagnoses through clinical and imaging examinations as well as histopathological studies. Treatment is surgical and involves mass resection. Recurrences are exceptional.
